# Biofilm feeding: Microbial colonization of food promotes the growth of a detritivorous arthropod

**DOI:** 10.3897/zookeys.577.6149

**Published:** 2016-04-05

**Authors:** Terézia Horváthová, Wiesław Babik, Ulf Bauchinger

**Affiliations:** 1 Institute of Environmental Sciences, Jagiellonian University, Gronostajowa 7, 30-387 Kraków, Poland

**Keywords:** Plant feeders, microorganisms, diet quality, bacterial gut community, growth

## Abstract

Feeding on plant material is common among animals, but how different animals overcome the dietary deficiencies imposed by this feeding strategy is not well understood. Microorganisms are generally considered to play a vital role in the nutritional ecology of plant feeding animals. Commonly microbes living inside animal bodies are considered more important, but recent studies suggest external microbes significantly shape plant-feeding strategies in invertebrates. Here we investigate how external microbes that typically form biofilm on primary plant material affect growth rates in a terrestrial isopod species *Porcellio scaber*. We experimentally manipulated the amount of biofilm on three different primary diet sources and quantified growth and survival of individuals that fed on food with either a small or large amount of biofilm. In addition, we tested how dietary manipulation shapes the composition of bacterial communities in the gut. The presence of visible biofilm significantly affected the growth of isopods: individuals that fed on the primary diet source with a large amount of biofilm gained more mass than individuals feeding on a diet with marginal biofilm. Diet also significantly affected the bacterial gut community. The primary diet source mainly determined the taxonomic composition of the bacterial community in the isopod gut, whereas the amount of biofilm affected the relative abundance of bacterial taxa. Our study suggests that terrestrial isopods may cope with low-quality plant matter by feeding on biofilm, with decomposition of plant material by organisms outside of the feeding organism (here a terrestrial isopod) probably playing a major role. Future investigations may be directed towards the primary diet source, plant matter, and the secondary diet source, biofilm, and should assess if both components are indeed uptaken in detritivorous species.

## Introduction

Plant material is the common food source for herbivorous and detritivorous animals, although it has low nutritional quality and is difficult to digest. While herbivores may not obtain enough nitrogen by feeding on living plants (Pierce and Berry 2011), the nutrient content of dead plant material is even lower for detritivorous species (Zimmer 2002a). Nevertheless, many herbivores and detritivores successfully consume plant material, but how they actually meet their nutritional requirements is still an unresolved question (but see Filipiak and Weiner 2014). Animals have employed different strategies to compensate for low-quality diet by simply processing more food per unit time (Woods 1999) or aggregate in social groups to have better access to food (Lihoreau et al. 2015). Herbivores benefit from mutualistic associations with symbionts that provide them with essential nutrients (e.g. aphids and amino acid requirements, Gunduz and Douglas 2009), and detritivorous species may profit from microbial colonization of dead plant material (Kautz et al. 2002; Zimmer 2002a; Zimmer et al. 2003). Another strategy used by wood boring beetles promotes a significant nutritional contribution of fungi that are ingested along with decomposed wood (Filipiak and Weiner 2014; Tanahashi et al. 2009). Beetles thus may cover their nutritional needs by feeding on an organism that itself lives on plant material.

Plant tissues are colonized by different microorganisms that often form multicellular complexes ranging from small aggregates to highly structured biofilms (Eberl et al. 2007). Biofilm can be defined as an assemblage of microbial cells that are enmeshed in a self-produced extracellular matrix (Davey and O’toole 2000). The biofilm matrix provides the mechanical stability of biofilms, mediates the adhesion to surfaces and association with interfaces, buffers biofilms from environmental conditions (Flemming and Wingender 2010) and may even serve as a nutrient source for biofilm-feeding animals due to its high content of polysaccharides (Lawrence et al. 2002). The formation and growth of biofilm may be affected by factors such as temperature, pH, nutrient availability on the substrate, or time (Else et al. 2003; Moghadam and Zimmer 2014; Rinaudi et al. 2006). The growth of biofilm is characterized by an initial rapid proliferation of microbial cells and increased microbial richness and diversity, which finally leads to the formation of a stable climax community (Davey and O’toole 2000; Douterelo et al. 2014). Microbial colonization of plant material also affects detritivore performance. Increased microbial activity and density may improve growth, survival and fecundity, and also enhance digestive processes in the gut (Kautz et al. 2000; Zimmer 1997b; 2002a). Although the role of biofilm as a nutritional source for detritivores has been recognized, the understanding of how diet shapes the microbial community composition within the digestive tract of a host animal is still poorly understood.

**Figure 1.**
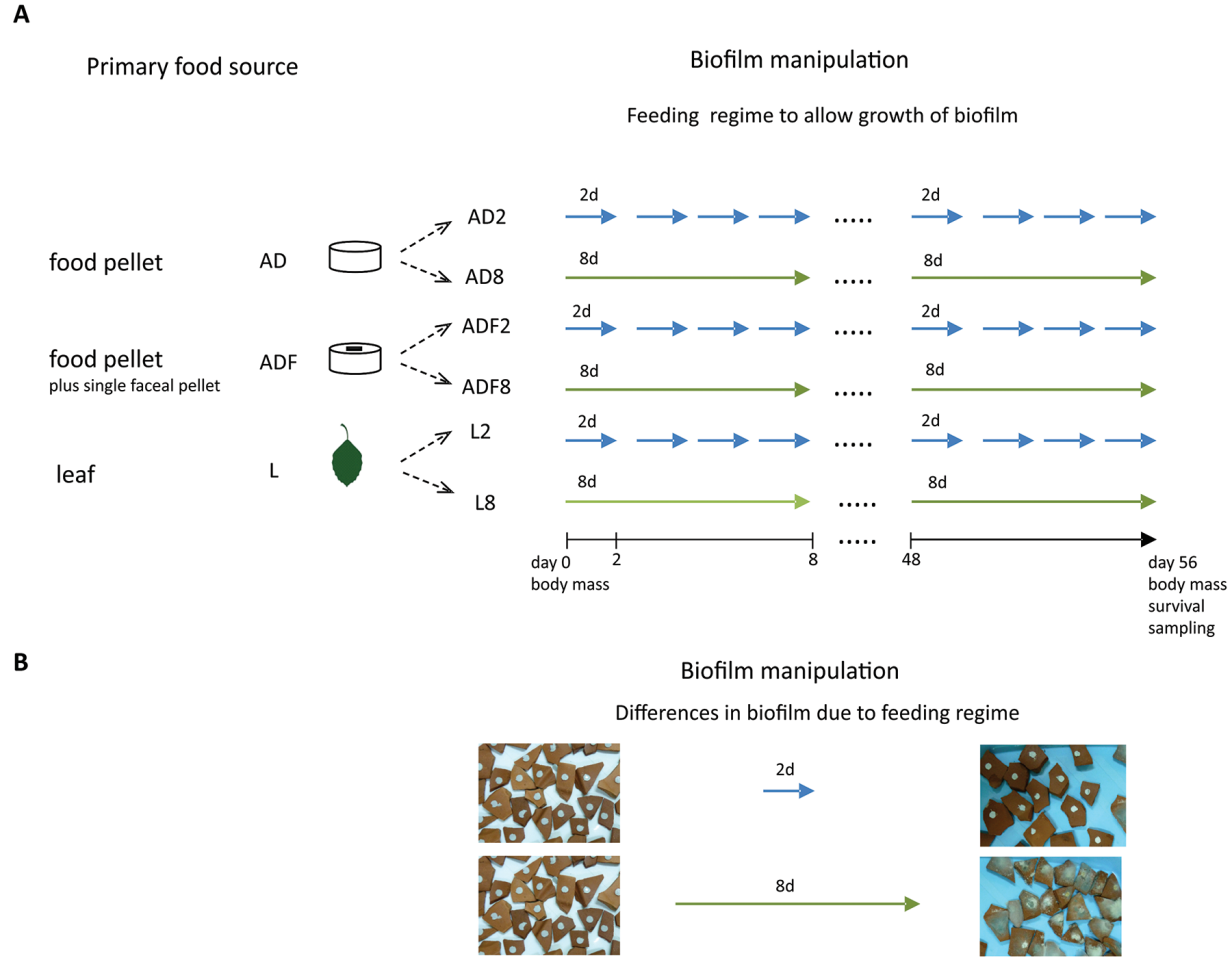
The scheme of the experimental design (**A**). The three primary diets: an artificial diet (AD), an artificial diet with a single faecal pellet of conspecific (ADF), and a single ash leaf (L) were split into two treatments (2d and 8d). With respect to these two treatments the food was renewed every 2 days or every 8 days to obtain food either substantially overgrown by biofilm or with marginal biofilm growth. Individuals were weighed at the beginning and the end of experiment. After final weighing, individuals were killed and the hindgut was dissected for molecular analyses. Part **B** shows fresh pellets of artificial diet (pictures left), which were renewed either every 2 days or every 8 days (pictures right).

To test for the general role of biofilm as an important food source for detritivorous isopods, we experimentally manipulated the amount of biofilm. We offered a primary diet source *ad libitum*, but we replaced the diet either after two days or after eight days, which allowed biofilm to develop on the primary diet source for different periods of time (see Figure 1B). Even after 8 days the primary food pellet was not substantially consumed indicating true *ad libitum* conditions with respect to primary food source for both, 2-day and 8-day groups. However, our feeding regime also resulted in a much larger amount of biofilm in the 8-day group from day two onwards, the day when the food pellet was replaced with a new one only in the 2-day treatment. We quantified the nutritional contribution of biofilm by determining the growth and survival rates of individuals of the terrestrial isopod species *Porcellio scaber* Latreille, 1804 that fed on three different primary diet sources, each overgrown by either low or high amount of biofilm. We also estimated microbial community composition in the gut for a subset of individuals by 16S metagenomics. We used the species *Porcellio scaber* which is considered to feed on plant material and is generally described as a detritivore, though it preferentially feeds on a diet inoculated with microbes (Ihnen and Zimmer 2008). We tested the following predictions: i) a primary diet source with a large amount of biofilm improves the growth and survival of individuals compared to a primary diet source with a small amount of biofilm, and ii) a similar amount of biofilm, regardless of the primary diet source, promotes similar patterns of isopod growth and survival. Bacterial communities of biofilm are mainly shaped by the type of substrate (Li et al. 2014) and the formation of biofilm is also characterized by the proliferation of attached microbial cells and by changes in species composition (effect of time, Sauer et al. 2002). Therefore, we further predicted that iii) the primary diet source or iv) the amount of biofilm on the primary diet source, affect bacterial gut communities in terms of taxonomic composition and the relative abundance of particular taxa.

## Materials and methods

### Animals and dietary manipulation

Specimens of woodlice (*Porcellio scaber*) were collected in the summer of 2013 in Kraków, Poland. The locality is situated in the courtyard of an old building, where isopods were found under rocks, bricks, trash or decaying trees. Adult individuals (236 males) were randomly chosen, weighed to the nearest 0.01 mg (Mettler Toledo XP26, Greifensee, Switzerland) and kept individually in separate boxes (52 × 48 mm, 100 ml) containing wet sand and a piece of clay pot. Individuals were equally assigned to three primary diets: an artificial diet (AD), an artificial diet with a single faecal pellet of a conspecific individual (ADF), and a single ash leaf (L). As a comparison to a natural isopod food source (leaves) which could potentially differ in quality, we chose an artificial diet that contains a similar amount of cellulose (30%) and has a well-defined composition (see Appendix). Leaves and the “artificial diet” were always offered *ad libitum*. These three diets were split into two treatments (2- and 8-day) to obtain food either substantially overgrown by biofilm (8-day) or with marginal biofilm growth (2-day; see Fig. 1A for the scheme of experimental design). When food was renewed every 2 days, only marginally visible biofilm could develop, whereas when food was renewed every 8 days, clearly visible biofilm overgrew the primary food source. The amount of biofilm could easily be verified visually (Fig. 1B) and was not quantified through other means. We expected that a longer incubation time (8 days) would promote the proliferation of microbial cells (Song and Leff 2006). Our experimental manipulation resulted in six experimental groups: AD2, AD8, ADF2, ADF8, L2 and L8. The boxes of the 8-day groups were opened every second day to mimic the disturbance in the 2-day groups during food changing. The ADF group was used to produce biofilm with a more natural (faeces-derived) bacterial community for terrestrial isopods, which often show coprophagous behaviour (Kautz et al. 2002). A single tiny faecal pellet (0.1-0.3 mg) was collected fresh from a box with individuals of *Porcellio scaber* not used in the experiment. This stock population was fed with ash leaves and was collected at the same site as experimental animals. The faecal pellet was used to ensure that microorganisms from faeces would colonize the food and produce a biofilm. The details of diet composition and diet preparation are presented in the Appendix. Sixty adult isopods were placed in experimental boxes sequentially in four blocks every week (240 in total). This gave forty isopods per experimental group. Individuals were weighed after four and eight weeks of growth.

### Analyses of growth and survival

All data were tested for normality of distribution and homogeneity of variance prior to analyses. To examine the effect of diet on body mass increase, a generalized linear mixed model (GLMM) was used with diet source (AD, ADF, L) and amount of biofilm (2 days, 8 days) as fixed factors, and the interaction term between the two factors. The block of animals was a random factor, and the initial body mass was a covariate. Body mass increase was calculated as the difference between the initial body mass and body mass after two months.

The GLIMMIX procedure was used to analyse differences in survival rates. The model included survival as a binary response variable (survived or died within eight weeks) with diet source and amount of biofilm as fixed factors. The block of animals was a random factor, and the initial body mass was a covariate. All statistical analyses were performed with the SAS 9.4 statistical software package (SAS Institute Inc., Cary, NC, USA).

### Bacterial community composition

Isopods were decapitated and the hindgut of each individual was dissected and stored in individual eppendorf tubes at -20 °C. Hepatopancreatic glands were not sampled as the bacterial community of *Porcellio scaber* is represented by resident symbiotic bacteria which are acquired from the environment during early life (Wang et al. 2007). Only two microbial species have been identified in the lumen of the hepatopancreas in *Porcellio scaber*: *Candidatus* Hepatoplasma and *Candidatus* Hepatincola, which according to phylogenetic analyses cluster with Mycoplasmatales (Mollicutes) and Rickettsiales (α-Proteobacteria), respectively (Wang et al. 2004a; Wang et al. 2004b). Total DNA was extracted from the guts of 36 individuals (n = 6 per experimental group), and biofilm was scraped from the food samples (n = 2 per group) using the Wizard genomic DNA Purification kit (Promega). Amplification and Illumina sequencing of 16S DNA fragments was done following established protocols (Caporaso et al. 2010). The V4 variable region of bacterial and archaeal 16 ribosomal DNA was PCR amplified using primers 515f and 806r. The samples were indexed using a 12 bp barcode added to the 5’ end of the 515f primer. For each sample, PCR reactions were done in triplicate and contained 1 µl of extracted DNA, 0.2 µM of each primer, 12.5 µl of PCR Multiplex kit (Qiagen) and PCR grade water added to a final volume of 25 µl. The PCR cycling programme was 94 °C for 15 min followed by 33 cycles of 94 °C for 45 s, 50 °C for 60 s, 72 °C for 90 s and a final extension step of 72 °C for 10 minutes. Two types of negative controls were included in each batch of PCR reactions: two extraction negative controls (to guard against contamination at the DNA extraction step) and two PCR negative controls (to control for contamination during PCR). Amplicon libraries were pooled at equimolar ratios and sequenced on an Illumina MiSeq machine, producing 150 bp reads.

Further analyses were carried out in QIIME (Caporaso et al. 2010). The reads were demultiplexed, quality controlled and trimmed, retaining only reads with at least 75 bp of consecutive high quality bases. To assign reads to Operational Taxonomic Units (OTUs), we followed the open OTU picking workflow in QIIME using Greengenes version 13_8 as the reference database. The resulting BIOM table contained 468,881 reads with a mean of 9768 ± (SD) 1666 reads per sample. Diversity analyses were based on rarefaction to 6630 reads per sample (n = 36), which corresponded to the smallest per sample read number in our dataset. Two measures of microbial diversity were used: phylogenetic β- and α-diversity. To determine the similarity of the bacterial community between the individuals and between food samples (β-diversity), we used UniFrac metric distances which are based on the fraction of branch lengths shared between two communities within a phylogenetic tree constructed from 16S rRNA gene sequences from all communities being compared (Lozupone and Knight 2005). We used a qualitative and quantitative phylogenetic measure of β-diversity; unweighted UniFrac considers only the absence or presence of lineages (i.e., taxonomic composition), while weighted UniFrac directly accounts for differences in relative abundances of lineages within communities (Lozupone et al. 2007). Permutational MANOVA (Anderson 2001) was used to test for the effect of our experimental variables (diet source, amount of biofilm) on bacterial communities using both unweighted and weighted UniFrac distance metrics. For the measure of phylogenetic α-diversity (only gut community), a phylogenetic diversity tree (PD) was used to test for differences between experimental conditions using a two-way ANOVA with diet source, amount of biofilm and diet source x amount of biofilm as explanatory variables. The permutational MANOVA was computed using the Adonis function in the VEGAN package implemented in R. ANOVA was calculated with the SAS 9.4 statistical software package (SAS Institute Inc., Cary, NC, USA).

## Results

### Growth and survival

**Figure 2.**
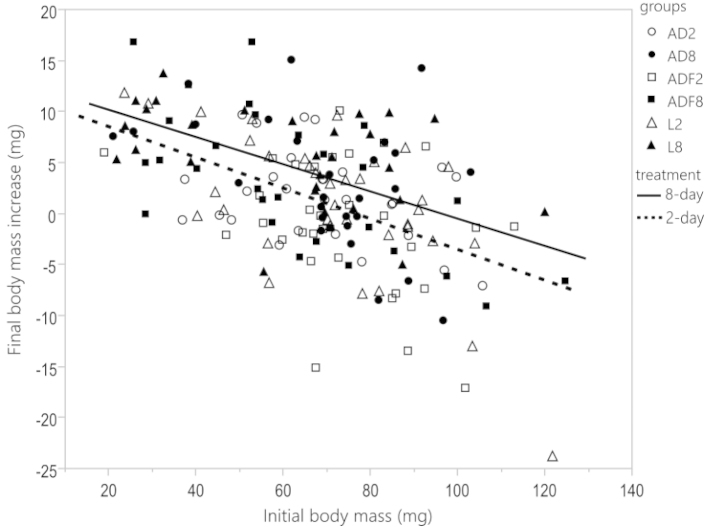
The relationship between initial body mass and the final body mass increase of woodlice feeding on the three primary diets (AD, ADF and L represent an artificial diet, an artificial diet inoculated with single faecal pellet, and leaves, respectively) either with small (2 days) or large (8 days) amount of biofilm. Regression lines represent the pooled data either for 2 or 8 days.

**Figure 3.**
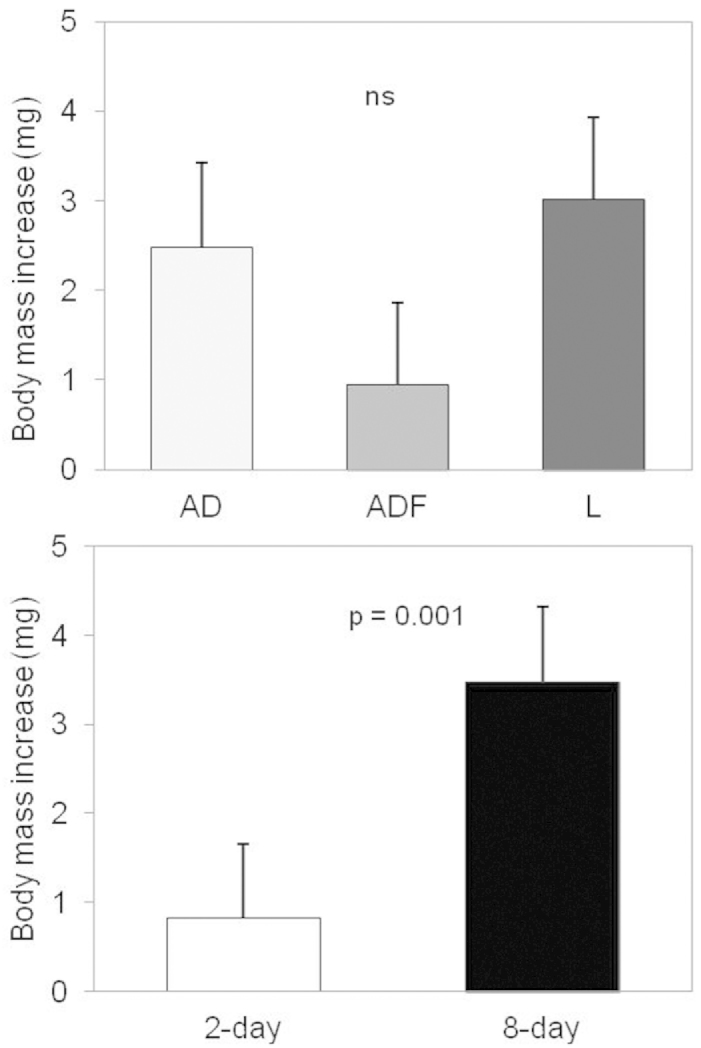
The effect of diet source (**A**) and amount of biofilm (**B**) on the final body mass increase of woodlice (least square means ± SE) after two months of growth (AD, ADF and L represent an artificial diet, an artificial diet inoculated with single faecal pellet, and leaves, respectively). Please note that isopods started at the average body mass of 68.5 mg.

Mean initial body mass did not differ between the six experimental groups (F_5,240_ = 1.4, p = 0.225) and was on average (±SD) 68±18 mg (AD2), 70±19 mg (AD8), 72±18 mg (ADF2), 64±23 mg (ADF8), 72±22 mg (L2) and 64±25 mg (L8), and had a negative effect on growth (Fig. 2, *F_1,169_* = 42.96, *p* < 0.0001). The final body mass increase differed significantly between the 2-day and 8-day groups (F_1,169_ = 10.76, p = 0.001). Individuals on 8-day biofilm diets had a higher increase in body mass than individuals feeding on a diet with 2-day biofilm (Fig. 2 and Fig. 3A). Primary diet (AD, ADF, L) and the block of animals did not have a significant effect on body mass increase (Fig. 3B, diet source: *F_2,169_* = 2.63, *p* = 0.075; block: *p* = 0.189). The interaction between diet source and amount of biofilm was not significant (*F_2,169_* = 0.47, *p* = 0.627).

The number of surviving individuals was relatively high (out of the initial 40: AD2 = 31, AD8 = 29, ADF2 = 31, ADF8 = 31, L2 = 36, L8 = 28). Survival did not differ between experimental groups (diet source: *F_2,230_* = 0.54, *p* = 0.58; amount of biofilm: *F_1,230_* = 2.91, *p* = 0.09; diet source x amount of biofilm *F_2,230_* = 1.5, *p* = 0.74). The initial body mass did not have a significant effect on survival (*F_1,230_* = 0.11, *p* = 0.74).

### Bacterial gut community composition

A total of 20 bacterial phyla and unclassified bacteria were detected in the guts of the isopod *Porcellio scaber* (n = 36). The majority of sequences belonged to *Proteobacteria* (84.1% of the classified sequences), *Bacteroidetes* (7.4%), *Actinobacteria* (3%), *Firmicutes* (1.9%), *Verrucomicrobia* (1.1%), *Cyanobacteria* (0.87%), *Tenericutes* (0.72%) and unclassified bacteria (0.7%). At the bacterial class level, 18%, 2.1% and 64% of total sequences represented *Alpha*-, *Beta*- and *Gammaproteobacteria*, respectively. In phylum *Bacteroidetes*, 2.6%, 2.1% and 2% represented *Flavobacteriia*, *Sphingobacteriia* and *Saprospirae*, respectively. Phylum *Actinobacteria* was represented only by the class *Actinobacteria* (order *Actinomycetales*). The dominant class of *Firmicutes* was *Bacilli* (1.88%). *Verrucomicrobia* was represented by the class *Verrucomicrobiae* (0.78%) and *Spartobacteria* (0.35%). The dominant classes of *Cyanobacteria* and *Tenericutes* were *Chloroplast* (0.8%) and *Mollicutes* (0.72%).

### The effect of dietary manipulation on bacterial composition in gut and on biofilm

The bacterial phylodiversity (measure of α-diversity) did not differ between gut samples (diet source: *F_2,29_* = 0.15, *p* = 0.863; amount of biofilm: *F_1,29_* = 1.58, *p* = 0.219; diet source x amount of biofilm: *F_2,29_* = 1.53, *p* = 0.233). The analysis of similarity (measure of β-diversity) showed that the taxonomic composition of gut bacteria was significantly shaped by the primary diet source (PERMANOVA: unweighted UniFrac, *p* = 0.001). When the relative abundance of lineages was accounted for, the effect of primary diet source on bacterial composition became weaker (PERMANOVA: weighted UniFrac, *p* = 0.057). The amount of biofilm significantly affected the relative abundance of taxa (weighted UniFrac, *p* =0.047) but not taxonomic composition (unweighted UniFrac, *p* = 0.268). This result suggests that the amount of biofilm affected the bacterial community quantitatively (relative abundance of taxa) rather than qualitatively (taxonomic composition). The largest differences in community structure between the 2-day and 8-day groups involved the bacterial phyla *Bacteroidetes* and *Proteobacteria*. Differences in taxonomic composition between experimental groups are presented in Table 1.

**Table 1.** Percentage of sequence reads for dominant bacterial phyla (i.e., operational taxonomic units) of individuals *Porcellio scaber* that fed on different diets (taxa which represented less than 0.01% of sequence reads were not included).

Bacteria phylum	Artificial diet	Artificial diet single faeces	Leaves	2-day biofilm	8-day biofilm
*Actinobacteria*	2.06	1.96	5.05	3.58	2.47
*Bacteroidetes*	8.97	8.12	5.24	11.25	3.63
*Cyanobacteria*	0.2	0.27	2.12	0.77	0.96
*Firmicutes*	3.8	1.76	0.1	1.55	2.23
*Proteobacteria*	83.28	85.16	83.95	79.96	88.3
*Tenericutes*	0.03	0.26	1.85	1.24	0.2
*Verrucomicrobia*	0.46	1.73	1.18	0.77	1.48
Other	1.09	0.51	0.67	0.7	0.64

Despite the small sample size (n = 2 per diet group), we tested for differences in the bacterial communities of the different biofilms. The analysis of similarity showed that taxonomic composition on biofilm was affected by the primary diet source (PERMANOVA: unweighted UniFrac, *p* = 0.002). When the relative abundance of bacterial taxa was accounted for, the bacterial community differed between the 2-day and 8-day groups, although the effect was weak (PERMANOVA: weighted UniFrac, *p* = 0.08).

## Discussion

Adult *Porcellio scaber* feeding on a diet overgrown by biofilm gained significantly more body mass than adults feeding on a diet with no visible biofilm. This finding was independent of the primary food source, i.e., the presence of a visible biofilm always promoted higher growth rates. The higher growth rate in association with the provisioning of a large amount of biofilm was also accompanied by changes in bacterial gut community composition. Individuals that fed on 8- or 2-day biofilm differed in relative abundance of bacterial lineages but not in taxonomic composition. Individuals that consumed different primary food sources (both AD and ADF) differed in bacterial taxonomic composition which was further supported by analyses of the biofilm samples. Our results strongly support the hypothesis that biofilm can be of high nutritional benefit for the detritivore isopod *Porcellio scaber*.

Leaf litter, which is a natural food source for detritivorous animals, is overgrown by biofilm composed of different fungal and bacterial species (Teuben and Roelofsma 1990) which can positively affect various life history traits. For example, high microbial activity of leaf litter positively affected the reproductive success and survival of various isopod species (Kautz et al. 2000; Rushton and Hassall 1983; Zimmer 2002b; Zimmer and Topp 1997). However, a positive impact of microbiota on isopod performance is not general. Some species such as *Oniscus asellus* may not depend on microorganisms when consuming low-quality detrital food sources (Zimmer and Topp 2000). In our study, survival did not differ between well-developed biofilm and marginal biofilm diets, but our experiment only lasted for two months, which might be too short to detect differences in survival rate based on the current sample size. Our results also show that faeces-colonizing microbiota did not increase the nutritive value of food as suggested by an earlier study (Hassall and Rushton 1982), questioning the nutritional role of coprophagy in terrestrial isopods (see also Kautz et al. 2002). Fungi are a common taxonomic group in biofilm associations and feeding on preferred fungal species increased the growth and reproduction of the Collembola species *Folsomia candida* and *Protaphorura armata* (Scheu and Simmerling 2004). We did not include fungi in our study since biodiversity of fungi is until today poorly known and the optimal DNA-based methods for its assessment are still debated (Kõljalg et al. 2013). The nutritional contribution of leaf litter biofilm has been generally attributed to the degradation of cellulose (Voriskova and Baldrian 2013; Zimmer and Topp 1997; 1999). Alternatively, the biofilm community may also provide limiting nutrients (Filipiak and Weiner 2014; Thompson et al. 2002; Zimmer and Topp 1998) or increase the consumption rate of feeding animals through indicating high-quality food sources (Zimmer et al. 2003). In our study, the greater mass increase of individuals feeding on diets with a high amount of biofilm suggests that the biofilm community improves the nutritional value of the primary food source. This may be facilitated indirectly through increased feeding rate of individuals and/or directly through digestion and utilization of microbial mass as an additional nutrient source. Thus, our results support a significant nutritional role of biofilm in the detritivore isopod *Porcellio scaber* (see also Zimmer and Topp 1998).

Diet is also considered to be one of the main factors determining the microbial gut community (Ley et al. 2008; Staubach et al. 2013). Our study showed that the bacterial community of the isopod gut was dominated by *Proteobacteria*, *Bacteroidetes*, *Actinobacteria* and *Firmicutes* which have also been identified as dominant phyla in various insect species, with *Proteobacteria* being predominant phyla in all insect guts studied so far (Jones et al. 2013; Yun et al. 2014). Terrestrial isopods can harbour microorganisms in two morphologically and functionally distinct parts of the digestive tract, the hepatopancreas and the hindgut (Zimmer 2002a). Whereas resident bacterial symbionts in the hepatopancreas possibly contribute to cellulose hydrolysis, the hindgut community of transient microbes and fungi might serve as a source of food-limited nutrients (Zimmer and Topp 1998). Two bacterial symbionts appear in the hepatopancreas, *Candidatus* Hepatoplasma and *Candidatus* Hepatincola, but never occur together in a single specimen and the percentage of aposymbiotic individuals varies across studied populations (10% in a German and 70% in a French population of *Porcellio scaber*, Zimmer 2006). The establishment and maintenance of a resident bacterial population in the hindgut is considered unlikely due to simple gut anatomy, the frequent renewal of the gut cuticle, and the short retention time of food (Kostanjšek et al. 2006). Furthermore, diversity in the gut bacterial community might also be affected by host identity (Dittmer et al. 2012), suggesting that not only diet but also some specific aspects of physiology or behaviour of the host may significantly shape the gut microbiota (Ye et al. 2014). The amount and variability of ingested food has been suggested to affect gut community composition; however, experimental demonstration is restricted to only two insect, two crustacean and three vertebrate species (Bertino-Grimaldi et al. 2013; Bolnick et al. 2014; Chandler et al. 2011; Dittmer et al. 2012; Turnbaugh et al. 2009). Wang et al. (2007) found that the bacterial community in the hepatopancreas of isopods differed between species living in semi-terrestrial, terrestrial and freshwater habitats. This suggests that the acquisition of hepatopancreatic symbionts in isopods might be the consequence of an evolutionary change in feeding habit. Our results show that the primary diet source significantly shaped the taxonomic composition of the bacterial community of the isopod gut, although it did not directly affect the growth rate. Since microbial community composition is better reflected by microbial activity, it is microbial biomass (i.e., nutrients) rather than activity that determines the importance of microbes for detritivore isopods. Because growth was mainly affected by the amount of biofilm which was expected to differ in total abundance of bacterial and fungal cells (Figure 1B, Song and Leff 2006), the quantity rather than the diversity of gut microbes affect isopod growth rates. In addition, different bacterial community structures in the 2-day and 8-day groups show that bacterial communities responded to our experimental manipulation (i.e., time for biofilm development on the primary diet source). Our results suggest that feeding on biofilm composed of a variety of bacterial taxa helps to meet the nutritional requirements of *Porcellio scaber* and thus enables it to subsist on a low-quality terrestrial diet. Fungi may likely contribute to this effect, however we did not quantify this contribution.

## Conclusion

A combination of experimental, molecular and life-history analyses revealed that biofilm may represent an important food source for the terrestrial isopod *Porcellio scaber*. Plant feeding animals may solve their nutritional dilemma by associations with micro-organisms within the digestive system that enhance the digestibility of plant material and/or act as a direct food source. Alternatively, as suggested here, these animals could feed on micro-organisms that grow on the plant material. Future studies may be directed towards the separation of the uptake of plant material from the consumption of biofilm growing on the plant material by different herbivorous and detritivorous species. Such an understanding may contribute to the ongoing discussion about the separation of herbivory and detrivory in nature (see also Farmer and Dubugnon 2009). Many of these species may actually be “biofilmivors” that achieve a nutritionally balanced diet through the utilization of biofilm.

Concerning the multi-organism nature of biofilm, future studies may benefit by covering a wider range of the taxa that compose biofilm, including protists and fungi. It would not be surprising if the varied decompositional potential of different plant taxa determine the value and importance of biofilm. Potentially, the ingested microorganisms themselves represent the main part of the processed food. Terrestrial isopods such as *Porcellio scaber* may rely much less on internal microbes to provide key enzymes, but rather take advantage of external microbes that predigest different resources which then become the primary food source.

## Appendix

The composition of artificial diet (Zimmer 1997a) modified after (Carefoot 1984):

Minimum diet (dry mass %): casein 15%, cellulose 30%, starch 25%, sucrose 10%, maltose 5%, glucose 5%, lactose 5%, di-Potassium hydrogen phosphate 1.15%, magnesium sulphate anhydrous 0.65%, copper chloride dihydrate 0.2%, sodium dihydrogen phosphatemonhydrate 0.45%, sodium chloride 0.2%, calcium hydrogenphosphate 0.65%, calcium lactate pentahydrate 1.55%, iron citrate 0.15%.

The preparation of diet:

A small amount of agar was sprinkled into boiling water in a glass beaker. The ingredients for minimum diet were added to the beaker while keeping the fluid warm. After stirring, the diet was poured into a sterile, plastic Petri dish and kept at 4 °C. Small pellets of artificial diet were cut out with plastic pipette tips.
